# Association between sleep quality and cancer-related cognitive impairment in patients with cancer: a meta-analysis

**DOI:** 10.3389/fneur.2026.1768687

**Published:** 2026-03-18

**Authors:** Zhenjuan Dang, Meirong Fan, Yao Zhang, Liyuan Lu, Jinling Gu, Caihong Zhang, Pingting Zhu

**Affiliations:** 1 School of Nursing, Faculty of Medicine, Yangzhou University, Yangzhou, Jiangsu, China; 2 Xinyi People’s Hospital Affiliated to Kangda College of Nanjing Medical University, Xuzhou, Jiangsu, China

**Keywords:** cancer-related cognitive impairment, chemotherapy brain, correlation, meta-analysis, sleep

## Abstract

**Objectives:**

This meta-analysis aimed to systematically evaluate and synthesize existing evidence to elucidate the association between sleep quality and cancer-related cognitive impairment (CRCI).

**Methods:**

PubMed, Embase, Scopus, Web of Science, China National Knowledge Infrastructure (CNKI), China Biology Medicine Disc (CBM), Wanfang data, and the VIP Journal Resource Integration Service Platform (VIP) were systematically searched inception to July 2025. The quality of the literature was evaluated using the Newcastle-Ottawa Scale and the Joanna Briggs Institute Cross-sectional Study Evaluation Checklist. The effect size *r* was transformed into Fisher’s *z*-values for statistical analysis. The combined analysis was performed using Fisher’s *z*-values and its standard error, and then the combined *z*-values were converted back to *r* for the discussion of the results. Statistical analyses were performed using Stata v.18.0.

**Results:**

A total of 4,037 articles were retrieved, and 13 articles (2,908 participants) were selected for further analysis. The meta-analysis based on the correlation coefficient *r* revealed a significant association between sleep quality and CRCI, with a correlation coefficient of *r* = −0.44 (95% CI: −0.55, −0.32). Sensitivity analysis confirmed the robustness of the (*r* = −0.47 to −0.40), and publication bias was not detected (*p* = 0.410). A supplementary meta-analysis of six studies using standardized regression coefficients further supported this association (*β* = −0.28, 95% *CI*: −0.34 to −0.22).

**Conclusion:**

Studies have found a moderate negative correlation between sleep quality and CRCI, suggesting that poorer sleep quality is associated with more severe cognitive impairment.

**Systematic review registration:**

PROSPERO RD420251090655.

## 1 Introduction

Cancer-related cognitive impairment (CRCI) refers to cognitive decline that occurs during cancer diagnosis, treatment, and rehabilitation. Common clinical manifestations include memory impairment, difficulty concentrating, slowed information processing, and impaired executive functions. CRCI, also known as chemotherapy-related cognitive dysfunction and commonly referred to as “chemotherapy or chemo brain,” has a pathogenesis that has not been fully understood. It involves factors such as inflammation, oxidative stress, and the neurotoxicity of chemotherapy (1), which has become one of the most persistent complications in cancer treatment (2). The incidence of CRCI varies among different types of cancer types, ranging from 15 to 75% and changes with the progression of the disease (3).

Sleep disorders are defined as clinical syndromes that involve disruptions of the sleep–wake cycle due to multifactorial etiologies, resulting in poor sleep quality or aberrant sleep-related behaviors (4). Among patients with cancer, sleep disorders exhibit a high prevalence, affecting approximately 30 to 88% of individuals across all disease stages, including initial diagnosis, progression, treatment, and recovery (5).

In recent years, research on the association between sleep quality and CRCI has focused primarily on neuropsychology, psycho-oncology, and behavioral medicine. From the perspective of inflammatory mechanisms, persistently activated immune cells release pro-inflammatory cytokines that can cross the blood–brain barrier and act on the central nervous system, participating in the regulation of the sleep–wake cycle. Simultaneously, these cytokines contribute to the formation of symptom clusters such as pain, fatigue, and mood disorders, which intertwine with cognitive decline, forming a complex pathological network of multi-symptom interactions (6). Wu et al. (7) demonstrated that in patients with cancer, the biopsychosocial symptom cluster of pain-fatigue-sleep disorder-psychological distress exhibits pronounced synergistic effects. Cognitive decline may precipitate negative emotions such as anxiety and depression, which when combined with the neurotoxic effects of chemotherapeutic agents, exacerbate sleep disorders. In contrast, chronic sleep disruption can alter impair neural repair mechanisms and metabolic clearance, thereby worsening cognitive deficits and establishing a self-perpetuating cycle of deterioration (8).

Regarding the specific association between sleep quality and CRCI, the currently research available research findings remain inconsistent. Although some studies have proposed additive or synergistic interactions between sleep disorders and CRCI, suggesting that sleep disturbances influence both the incidence and severity of cognitive impairment (9), others have reported no significant association (10). To reconcile these discrepancies, this study employed a meta-analytic approach to quantitatively synthesize existing evidence, systematically evaluating the correlation between sleep quality and CRCI, with the goal of reaching a consistent conclusion and providing new insights for further research on the relationship between sleep and CRCI.

## 2 Methods

This study was conducted according to the Preferred Reporting Items for Systematic Reviews and Meta-analyzes (PRISMA) (11). The systematic review protocol was registered with PROSPERO (Registration NO.: CRD420251090655). Systematic Review Registration: https://www.crd.york.ac.uk/prospero/view/CRD420251090655.

### 2.1 Literature search

Two reviewers independently executed a systematic literature retrieval in eight major databases from inception through July 2025, including PubMed, Embase, Scopus, Web of Science, China National Knowledge Infrastructure (CNKI), China Biology Medicine Disc (CBM), Wanfang data, VIP Journal Resource Integration Service Platform (VIP). The search combined Medical Subject Headings (MeSH) with free-text terms. Keywords and a detailed search strategy example of using PubMed include: ((Cognition[MeSH Terms]) OR (“Cognition Disorders”[Title/Abstract] OR “Cognitive Dysfunction”[Title/Abstract] OR “Cognitive Deficit”[Title/Abstract] OR “cognitive impairment”[Title/Abstract] OR “cognitive deficit”[Title/Abstract] OR “cognitive problem”[Title/Abstract] OR “cognitive defect”[Title/Abstract]))AND((Neoplasms[MeSH Terms]) OR (“Neoplasms”[Title/Abstract] OR “Carcinoma”[Title/Abstract] OR “cancer”[Title/Abstract]))AND((sleep[MeSH Terms]) OR (“Sleep Disorders”[Title/Abstract] OR “Insomnia”[Title/Abstract] OR “sleep disturbance”[Title/Abstract] OR “sleep disturb”[Title/Abstract] OR “sleep complaint”[Title/Abstract])). Database-specific syntax adaptations were implemented for each platform (see Supplementary Table S1 for details). Supplementary search measures: searching of reference lists of relevant reviews.

### 2.2 Inclusion and exclusion criteria

#### 2.2.1 Inclusion criteria

The following studies were included: (1) observational studies, including cohort studies, case–control studies, and cross-sectional studies; (2) studies in Chinese and English; (3) studies including patients who met the criteria for cancer diagnosis; (4) studies in which research variables were sleep quality and CRCI; and (5) studies reporting the correlation between the two variables, the effect indicator was the *r*-value and the data was complete.

#### 2.2.2 Exclusion criteria

The following studies were excluded: (1) reviews, case reports, conference abstracts, comments/commentaries; (2) studies with republished; and (3) studies for which the methodological quality evaluation was deemed of poor quality.

### 2.3 Literature screening

All retrieved records were managed using EndNote X9, with removal of duplicate studies conducted using automated algorithms followed by manual verification. Two researchers independently performed the initial screening by evaluating titles and abstracts against predetermined eligibility criteria. Publications meeting preliminary requirements advanced to full-text assessment, during which researchers independently conducted comprehensive review, assessment, and documentation of inclusion/exclusion rationales. Inter-rater discrepancies were resolved through consensus discussions, with unresolved disagreements were arbitrated by a senior researcher.

### 2.4 Data extraction

Two researchers independently extracted data using standardized forms, including: (1) literature characteristics (author, year, study type, etc.); (2) demographic data (sample size, age, sex, level of education, etc.); (3) methodological information (assessment tools, etc.); and (4) outcome indicators (correlation coefficients, standardized regression coefficient).

### 2.5 Assessment of literature quality

The methodological quality of cross-sectional studies was evaluated using the Joanna Briggs Institute (JBI) checklist for analytical cross-sectional studies. Studies were classified as “high quality” if they met criteria 1, 3, 4, 5, 6, 7, and 8 (7 of 8 total items). Studies meeting ≤4 criteria were categorized as low quality. For cohort and case–control studies, the Newcastle-Ottawa Scale (NOS) was used, which evaluates the quality of the study in three domains: selection of study groups, comparability of cohorts, and evaluation of results. The NOS assigns a maximum of 4, 2, and 3 points, respectively, yielding a total score of 9. The studies were stratified as low quality (<4 points), moderate quality (4 to 6 points), or high quality (7 to 9 points). Two independent investigators conducted all quality assessments, with discrepancies resolved by discussion or consultation with a third reviewer.

### 2.6 Statistical analysis

To ensure consistency in interpreting the direction of the effect sizes, these were uniformly defined as follows: a negative effect size indicated that “more severe sleep problems were associated with greater cognitive impairment.” If the original study variables were in the opposite direction, the sign of the reported effect size was reversed. The standardized coefficient *β* and the correlation coefficient *r* extracted as effect sizes were subjected to separate meta-analyses. For the meta-analysis, standardized coefficients *β* were directly pooled for effect size synthesis. Spearman’s correlation coefficients were converted to *r*-values using Equation 1. When analyzing correlation coefficients *r*, these were first transformed into Fisher’s *z*-values using Equation 2, followed by the calculation of the corresponding *V*-value of each study and standard error (SE) using Equations 3, 4. The pooled Fisher’s z value was then converted back to the pooled correlation coefficient *r* using Equation 5 (12, 13).

Heterogeneity was assessed using the chi-square test, with the following criteria: if *p* ≥ 0.1 and *I*^2^ < 50%, it indicated low heterogeneity among studies, and a fixed-effects model was employed for the meta-analysis; if *p* < 0.1 or *I*^2^ ≥ 50%, a random-effects model was used. If significant heterogeneity was detected, subgroup analyses and regression analyses were conducted to identify its sources. Sensitivity analyses were performed by sequentially excluding individual studies to evaluate the robustness of the results. Funnel plots were generated, and Egger’s test was applied to assess publication bias. All analyses were performed using Stata v. 18.0, with statistical significance set at *p* < 0.05.


$$r=\mathrm{2sin}(r_{s}\pi /6)$$ (1)

$${\text{Fisher}}^{’}s\;z=0.5\times \ln((1-r)/(1+r))$$ (2)

$$V_{z}=1/(n-3)$$ (3)

$$\mathrm{SE}=\sqrt{\mathrm{Vz}}$$ (4)

$$\text{Summary}\;r=(e^{2Z}-1)/(e^{2Z}+1)$$ (5)

## 3 Results

### 3.1 Literature search results

**Figure 1:**
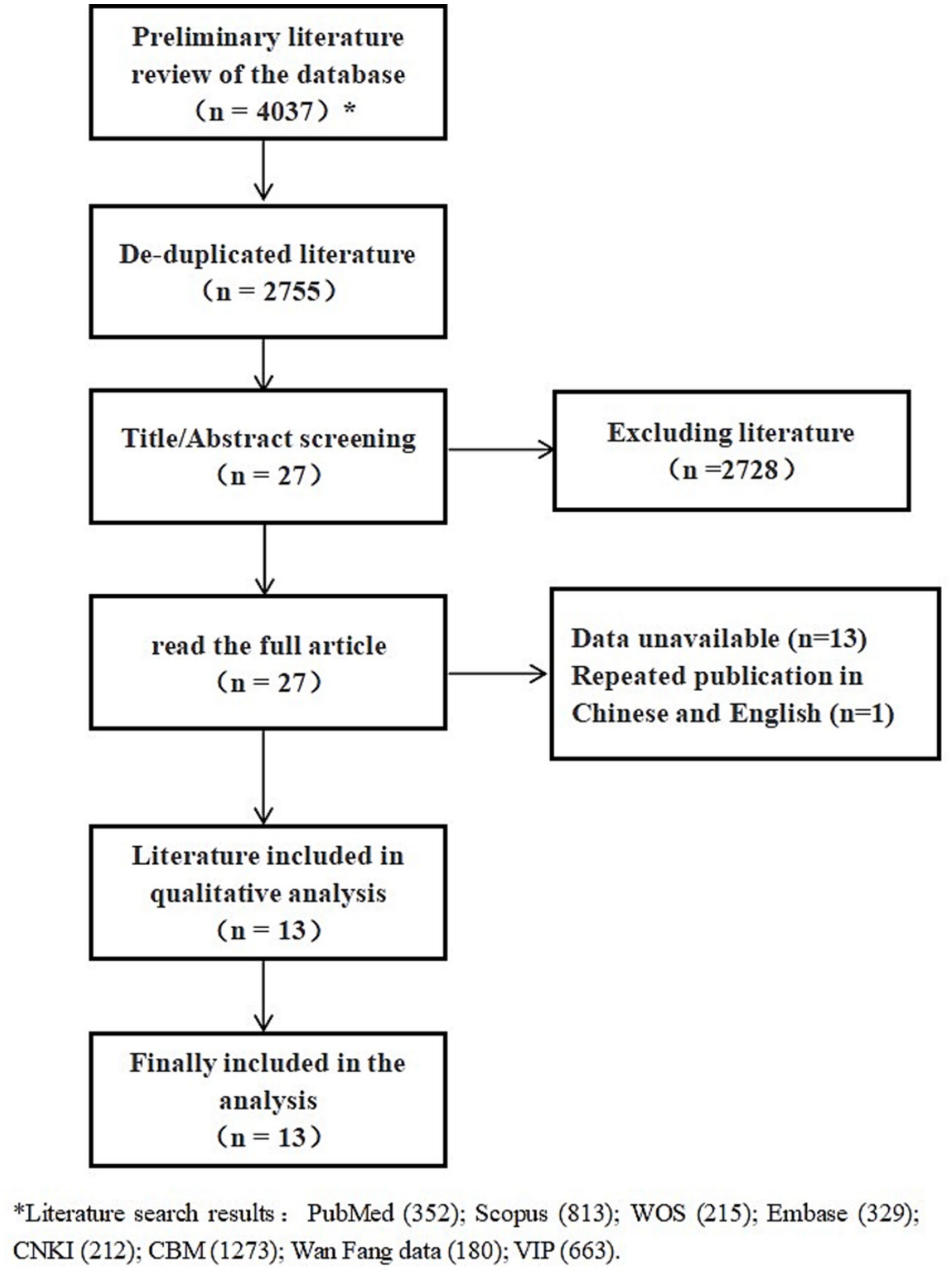
Flowchart of literature screening.

A total of 594 duplicate articles were automatically removed using Endnote, followed by the manual removal of an additional 688 duplicates studies. After screening titles and abstracts, 2,728 irrelevant articles were excluded. Upon full-text review, 13 articles were excluded due to the inability to extract *r*, and a duplicate publication in both Chinese and English was removed. Ultimately, 12 cross-sectional studies and one cohort study were included in the analysis. The study selection process is illustrated in Figure 1.

### 3.2 Basic characteristics and quality assessment of included literature

#### 3.2.1 Basic characteristics

This study included 13 observational studies, comprising 12 cross-sectional studies (14–25) and one cohort study (26), with a total sample size of 2,908 participants. The studies originated primarily from China (8 studies), followed by the United States (3 studies), Canada (1 study), and Australia (1 study). The study populations consisted predominantly of patients with breast cancer (7 studies), with additional cohorts including patients with lung cancer (2 studies), primary liver cancer (1 study), head and neck cancer (1 study), bone cancer (1 study), and hematological malignancies (1 study). The sample sizes ranged from 30 to 741, with mean patient ages spanning 23.7 to 67.8 years. Sleep quality was assessed using the Pittsburgh Sleep Quality Index (PSQI; 12 studies) and the Insomnia Severity Index (ISI; 1 study). Cognitive function was evaluated using instruments such as the Functional Assessment of Cancer Therapy-Cognitive Function (FACT-Cog; 7 studies) and the Montreal Cognitive Assessment et al. (Others; 6 studies). Detailed characteristics are presented in Supplementary Table S2.

#### 3.2.2 Quality assessment results

Quality assessment was assessed using the JBI checklist for cross-sectional studies and the NOS for cohort studies. The evaluation indicated that the overall quality of the included studies was satisfactory, with no low-quality studies identified. Detailed quality assessment results are provided in Tables 1, 2.

**Table 1:** Quality assessment of included studies (JBI cross-sectional studies checklist).

Author/year	1	2	3	4	5	6	7	8	Results
Wu, 2024 (14)	Y	Y	Y	Y	Y	N	Y	Y	High
Fang, 2016 (15)	Y	N	Y	Y	Y	Y	N	Y	Medium
Meng, 2020 (16)	Y	Y	Y	Y	N	N	N	Y	Medium
Henneghan, 2018 (17)	Y	N	Y	Y	Y	Y	Y	Y	High
Gu, 2025 (18)	Y	N	Y	Y	N	N	Y	Y	Medium
Chen, 2024 (19)	Y	N	Y	Y	Y	Y	Y	Y	High
Zhang, 2020 (20)	Y	N	Y	Y	Y	Y	Y	Y	High
Rogers, 2008 (21)	Y	Y	Y	Y	Y	N	N	Y	Medium
Liu, 2024 (22)	Y	Y	Y	Y	Y	N	Y	Y	High
Chen, 2008 (23)	Y	N	Y	Y	Y	N	Y	Y	Medium
Hutchinson, 2021 (24)	Y	Y	Y	Y	N	N	N	Y	Medium
Xu, 2024 (25)	Y	N	Y	Y	Y	Y	Y	Y	High

1. Whether the inclusion criteria of the sample are clearly defined; 2. Whether the study subjects and study sites are described in detail; 3. Whether the measurement methods of exposure factors are reliable and valid; 4. whether there are objective and consistent criteria for the definition of diseases or health problems; 5. Whether confounding factors are identified; 6. Whether measures are taken to control confounding factors; 7. Whether the measurement methods of the outcome indicators have reliability and validity; 8. Whether the data analysis methods were appropriate.

**Table 2:** Quality assessment of included studies (NOS scale).

Author/year	Study subject selection				Comparability between groups	Outcome measures			Scoring	Results
	1	2	3	4	5	6	7	8		
Garland, 2022 (26)	1	1	1	1	2	1	1	0	8	High

1. Representativeness of exposure; 2. Selection of non-exposed group; 3. Determination of exposure factors; 4. Confirm that no outcome events occurred at the beginning of the study; 5. The comparability of the exposed and non-exposed groups was considered in the design and statistical analysis to control for confounding factors; 6. Adequacy of outcome evaluation; 7. Whether the duration of follow-up was sufficient; 8. Whether follow-up was adequate in the exposed and non-exposed groups.

### 3.3 Results of the meta-analysis

#### 3.3.1 Meta-analysis of the correlation coefficient between CRCI and sleep quality

**Figure 2:**
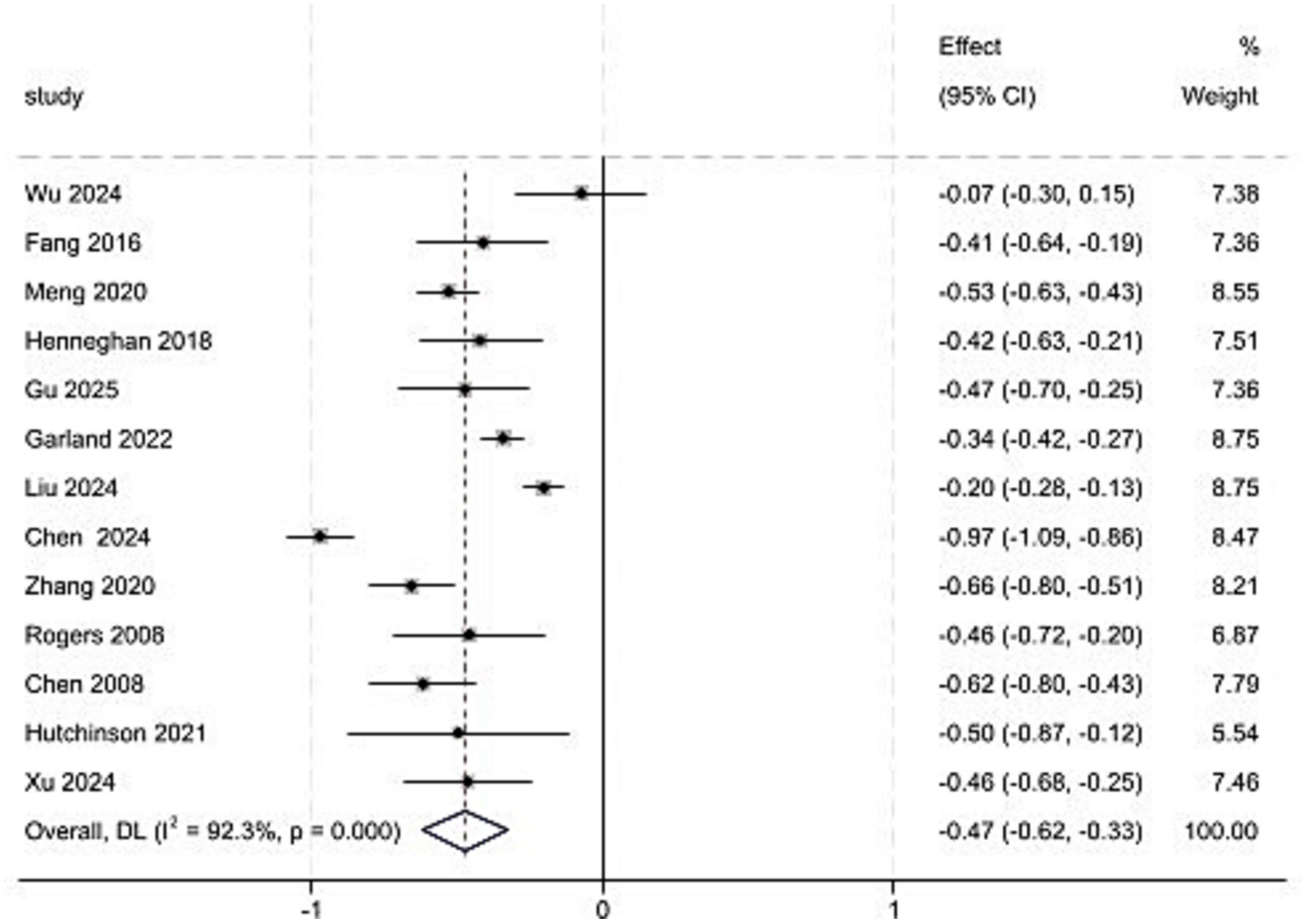
Meta-analysis of the correlation between CRCI based on *r* value and sleep quality.

**Figure 3:**
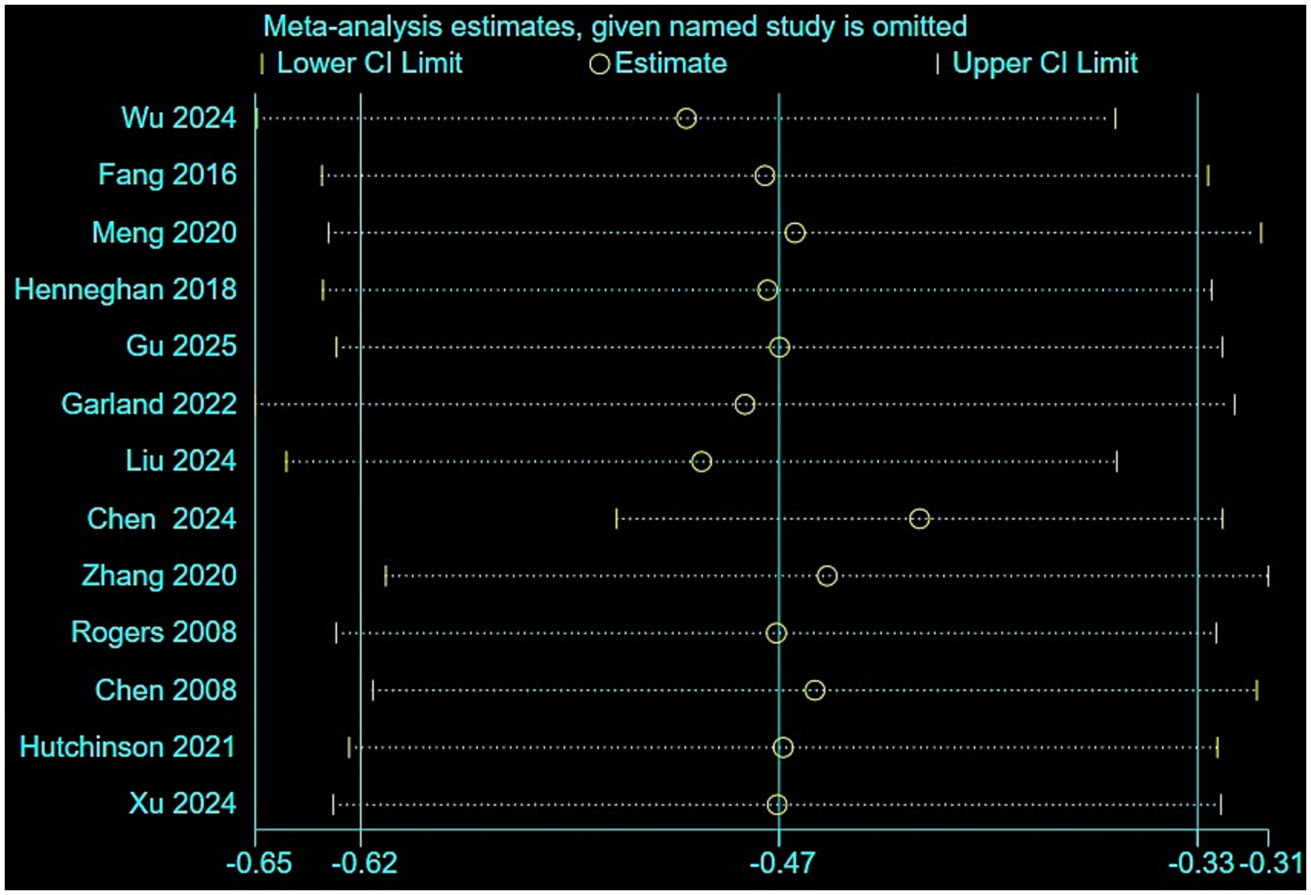
Sensitivity analysis of the correlation between CRCI and sleep quality.

A total of 13 studies were included in the analysis. The results of the heterogeneity test showed *p <* 0.001, *I*^2^ = 92.3%, and a random-effects model was employed for the meta-analysis. The results indicated that the correlation coefficient between CRCI and sleep quality was *z* = −0.47 (95% *CI*: −0.62 to −0.33), which converted to *r* = −0.44 (95% *CI*: −0.55 to −0.32) (Figure 2). Sensitivity analysis was conducted by sequentially excluding individual studies (Figure 3). The sensitivity analysis demonstrated stable results (*z* = −0.51 to −0.43), with converted values *r* = −0.47 to −0.40.

#### 3.3.2 Results of the subgroup analysis of the correlation coefficients between CRCI and sleep quality

Subgroup analyses were conducted based on region, CRCI measurement tools, diseases, effect size statistical methods, sex, treatment (Table 3). (1) Region. The pooled effect size in studies from China (*z* **=** −0.54, *r* **=** −0.49, *p* = 0.000) was stronger than that in studies from the United States (*z* **=** −0.32, *r* **=** −0.31, *p* **=** 0.035), though the inter-group difference did not reach statistical significance (*p* **=** 0.299). (2) CRCI measurement tools. Among tool subgroups, the pooled effect size for objective measurement tools was *z* **=** −0.27, *r* **=** −0.26, *p* **=** 0.013, while that for subjective measurement tools was *z* **=** −0.51, *r* **=** −0.47, *p* = 0.000, with no statistically significant difference between the two groups (inter-group heterogeneity *p* **=** 0.277). However, further stratification by specific measurement tools revealed significant inter-group differences (*p* **=** 0.008). (3) Disease. Among patients with breast cancer, the pooled correlation coefficient was *z* **=** −0.48, *r* **=** −0.45, *p* = 0.000. In other cancer types, the pooled correlation coefficient was *z* **=** −0.44, *r* **=** −0.41, *p* = 0.180, while inter-group difference did not reach statistical significance (*p* **=** 0.785). (4) Effect size statistical methods. The pooled effect size for the Spearman’s group was *z* **=** −0.26, *r* **=** −0.25, *p* **=** 0.028, whereas that for the Pearson’s group was *z* **=** −0.51, *r* **=** −0.47, *p* **=** 0.000, indicating a significant negative correlation, though no inter-group difference was observed (*p* **=** 0.231). (5) Sex: In studies that included only female patients, the pooled correlation coefficient between sleep disorders and CRCI was *z* **=** −0.47, *r* **=** −0.44, *p* **=** 0.000, whereas in studies that included male and female patients, it was *z* **=** −0.46, *r* **=** −0.43, *p* **=** 0.066, while inter-group difference did not reach statistical significance (*p* **=** 0.972). (6) Treatment. Among patients who had undergone chemotherapy, the pooled correlation coefficient between sleep disorders and CRCI was *z* **=** −0.49, *r* **=** −0.45, *p* **=** 0.000, while in those who had not received chemotherapy, it was *z* **=** −0.37, *r* **=** −0.35, *p* **=** 0.524, though no inter-group difference was observed (*p* **=** 0.265).

**Table 3:** Subgroup analysis of the correlation coefficient between CRCI and sleep quality.

Subgroups	Studies	Correlation coefficient (95%CI)	*I^2^* (*%*)	*P*
All	13	−0.47 (−0.62, −0.33)	92.3	0.000
(1) Region				0.299
United	3	−0.32 (−0.56, −0.07)	70.1	0.035
China	8	−0.54 (−0.76, −0.33)	94.9	0.000
Canada	1	−0.34 (−0.42, −0.27)	0.0	/
Australia	1	−0.50 (−0.87, −0.12)	0.0	/
(2) Types of measurement tools				0.277
Subjective measurement tools	11	−0.51 (−0.67, −0.35)	93.1	0.000
Objective measurement tools	2	−0.27 (−0.67.0.12)	83.9	0.013
(3) CRCI measurement tools				0.008
MoCA	1	−0.07 (−0.30, 0.15)	0.0	/
MMSE	1	−0.47 (−0.70, −0.25)	0.0	/
EORTC-CF	2	−0.52 (−0.73, −0.32)	48.6	0.163
PRMQ	1	−0.53 (−0.63, −0.43)	0.0	/
PCI	2	−0.71 (−1.24, −0.17)	95.1	0.000
CFQ	1	−0.34 (−0.42, −0.27)	0.0	/
FACT-Cog	4	−0.44 (−0.70, −0.18)	91.2	0.000
PCA	1	−0.50 (−0.87, −0.12)	0.0	/
(4) Diseases				0.785
Breast	6	−0.48 (−0.76, −0.21)	96.6	0.000
Others	7	−0.44 (−0.53, −0.35)	32.5	0.180
(5) Effect size statistical methods			0.231	
Spearman	2	−0.26 (−0.64,0.12)	79.2	0.028
Pearson	11	−0.51 (−0.67, −0.35)	93.2	0.000
(6) Male/female				0.972
Women	5	−0.47 (−0.82, −0.12)	97.2	0.000
Men and women	8	−0.46 (−0.55, −0.38)	47.3	0.066
(7) Treatment				0.265
Chemotherapy	9	−0.49 (−0.69, −0.29)	94.6	0.000
Non-chemotherapy	4	−0.37 (−0.43, −0.30)	0.0	0.524

#### 3.3.3 Meta-regression analysis results of the correlation coefficient between CRCI and sleep quality

A random-effects meta-regression analysis was conducted to examine the influence of sample size and mean patient age on the overall effect size. The results indicated that neither the sample size (regression coefficient = 0.000, *p* = 0.491) nor the mean patient age (regression coefficient = −0.004, *p* = 0.542) were significant predictors of the effect size. The estimated residual heterogeneity variance τ^2^ was 0.049, suggesting the presence of other unexplained heterogeneity between studies after controlling for these two variables.

#### 3.3.4 Meta-analysis of standardized regression coefficients of CRCI and sleep quality

**Figure 4:**
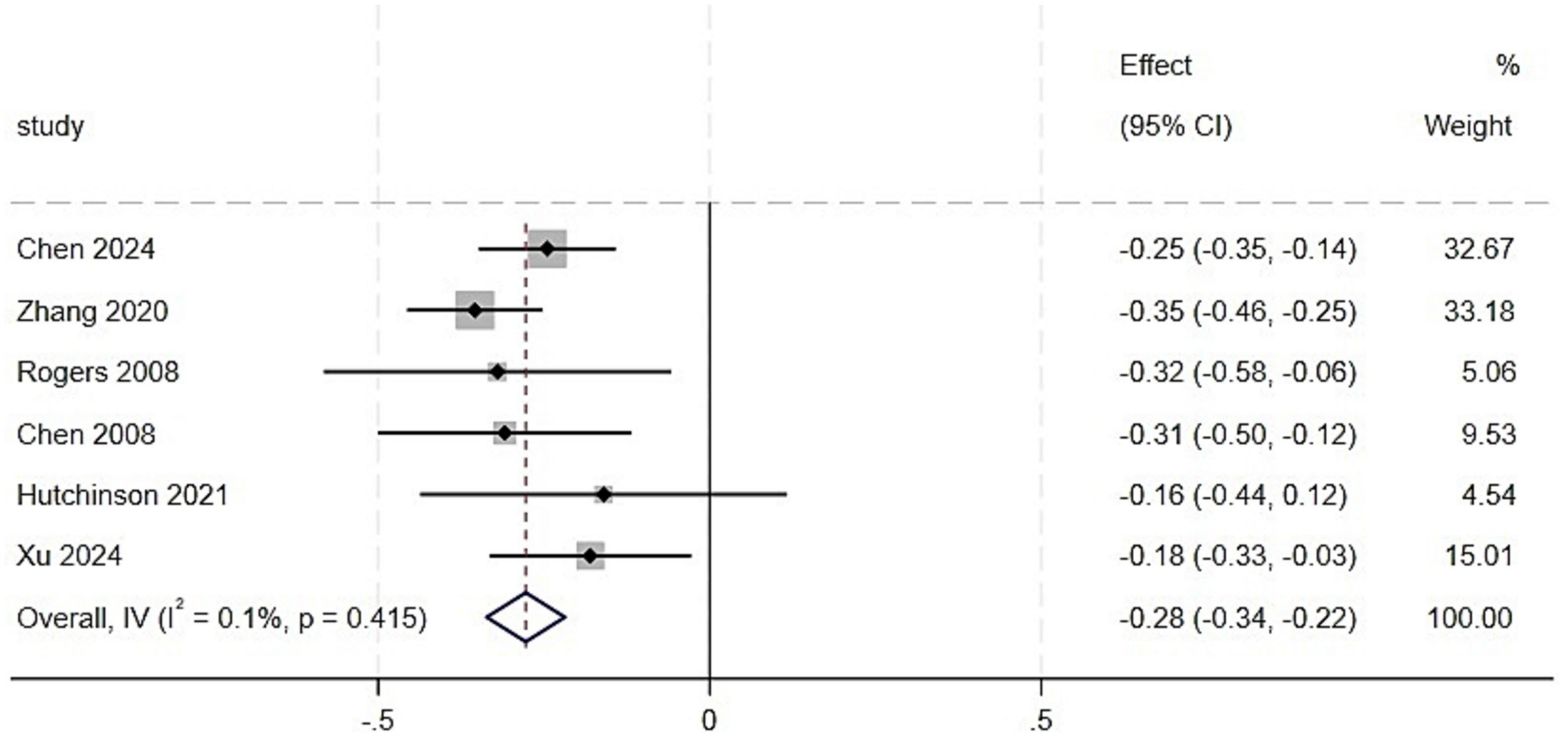
Meta-analysis of the correlation between CRCI based on standardized regression coefficients (*β*) and sleep quality.

For the six studies that reported standardized regression coefficients (*β*), a separate meta-analysis was performed (Figure 4). The heterogeneity among the studies was extremely low (*I*^2^ = 0.1%, *p* = 0.415), and a fixed-effects model was employed for the meta-analysis. The results revealed a significant negative association between sleep quality and CRCI after controlling for relevant confounding factors, with a pooled standardized coefficient of *β* = −0.28 (95% *CI*: −0.34 to −0.22, *p* < 0.001).

#### 3.3.5 Publication bias test

**Figure 5:**
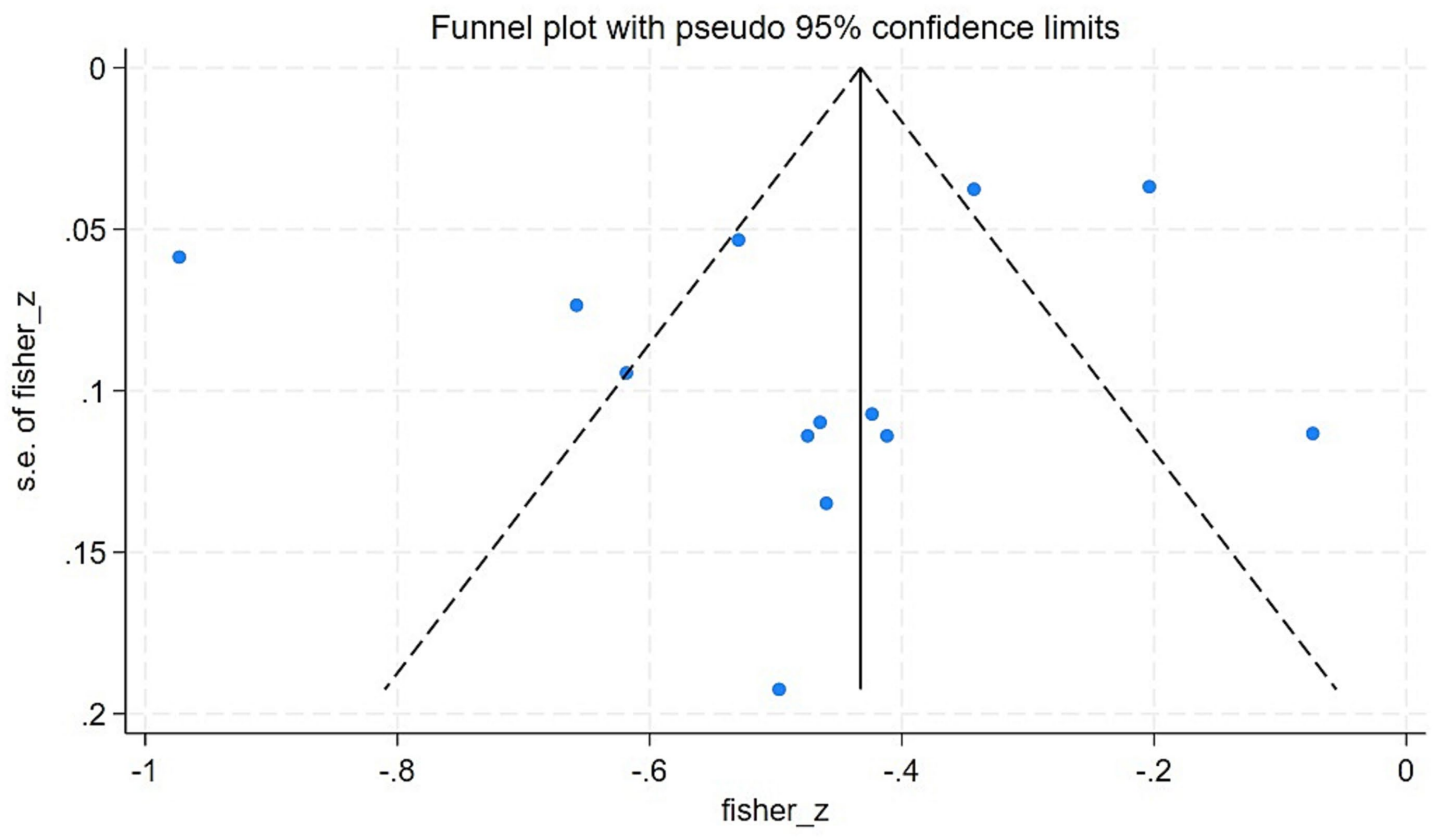
Funnel plot of the meta-analysis on the correlation between CRCI and sleep quality.

For the 13 studies based on *r*-values, Begg’s test did not indicate significant asymmetry (*z* = 0.06, *p =* 0.951). Egger’s test also did not show significant differences (*t* = −0.86, *p* = 0.410). In general, the statistical tests did not detect significant publication bias. Visual inspection of the funnel plot demonstrated that most of the study points were symmetrically distributed around the size of the pooled effect, indicating general symmetry (Figure 5). Due to the limited number of studies (*n* = 6) reporting *β*-values, no bias test was conducted.

## 4 Discussion

This study systematically investigated the correlation between sleep quality and CRCI in patients with cancer through meta-analysis. The results demonstrated a moderate negative correlation between sleep quality and CRCI (*r* = −0.44), and sleep quality was associated with CRCI after controlling for confounders (*β* = −0.28). These findings extend the well-established “sleep-cognition” association observed in the general population to the vulnerable group of cancer survivors (27).

The biological plausibility of our findings can be explained in terms of neuroinflammation and immune regulation. In the normal population, sleep disorders affect cognitive function by upregulating proinflammatory cytokines (such as IL-1β, IL-6, TNF-*α*), whereas the levels of brain-derived neurotrophic factor and synaptic plasticity-related proteins were downregulation (28). In patients with cancer, chemotherapy itself can induce similar neuroinflammatory responses and synaptic dysfunction. Sleep disorders may have a synergistic effect with chemotherapy to further aggravate the central neuroinflammatory environment, leading to changes in blood–brain barrier permeability and myelin damage, thus amplifying the severity of CRCI (29).

In addition, as sleep is a critical period for the systematic removal of metabolic waste from the brain, sleep disorders can lead to the accumulation of neurotoxic substances and accelerate cognitive decline. Drijver et al. (30) systematically reviewed the association between sleep disorders and neurocognitive function and explored the potential positive impact of sleep interventions on neurocognitive function in cancer survivors. Studies have indicated that poor sleep quality is associated with neurocognitive decline, which is consistent with the results of this study.

For the regional subgroup, the pooled effect size in the Chinese group was higher than that of the United States group, which may be attributed to cultural background and economic factors. For the CRCI measurement tool subgroup, the effect size of subjective measurement tools was higher than that of the objective measurement tool groups, although the difference between groups did not reach statistical significance. This result might be partially limited by the inclusion of only two studies in the objective tool group with a small sample size, leading to insufficient statistical power. Some studies suggest that subjective cognitive complaints may be associated with tau protein deposition in the parietal lobe, whereas objective memory decline is more related to hippocampal tau deposition (31), indicating that subjective cognitive function might reflect an earlier-stage or different mechanisms of pathological changes in the brain, potentially preceding cognitive impairment detectable by objective tests. This provides a potential neurobiological basis for explaining the differences in association strength between subjective and objective tools. Although the number of studies included in this study using objective CRCI measurements was limited, making it difficult to completely rule out chance effects, the diversity of measurement tools likely represents one of the main sources of the high heterogeneity observed in this study. In particular, when further stratified by specific assessment tools, significant differences emerged between different subgroups of tools. Other studies have reported that the prevalence of cognitive impairment after breast cancer chemotherapy ranges from 0 to 83%, with this substantial variation primarily driven by the choice of assessment tools (32). The findings suggest that subjective CRCI measurement tools should be prioritized in clinical practice. Currently, the most widely used subjective CRCI measurement tool in clinical settings is FACT-Cog (33). This study demonstrated that using the PCI subscale of the FACT-Cog questionnaire achieved higher sensitivity (−0.71 vs. − 0.44). The overall association strength between sleep disorders problems and cognitive impairment was similar in patients with breast cancer and in patients without non-breast cancer. However, high heterogeneity was observed within breast cancer studies compared to non-breast cancer populations, a finding that corresponds with the sex-based subgroup analysis (female/breast cancer). This suggests the unique characteristics of breast cancer populations and the individual heterogeneity of CRCI as well as the complexity of pathological mechanisms (34). Future research on CRCI in breast cancer should employ more specific population stratification. The statistical methods for effect sizes (Spearman’s vs. Pearson’s correlation coefficients) were not the primary source of heterogeneity among studies. This suggests that in meta-analyses, when some studies reported only Spearman’s correlation coefficients, combining them with Pearson’s correlation coefficients may not introduce significant bias. Whether patients received chemotherapy did not significantly explain the variation in effect sizes across studies. The high heterogeneity in this study was almost entirely concentrated within the chemotherapy-treated patient group. Combined with subgroup analysis by disease type, the high heterogeneity in the chemotherapy group overlapped substantially with that in the breast cancer group. Thus, the observed overall high heterogeneity was unlikely to stem from the isolated influence of any single factor.

The meta-regression results analysis indicates that the continuous variables (sample size and mean patient age) cannot account for the variation in effect sizes. This finding aligns with the results of the subgroup analysis, where broad categorical variables such as region, treatment, and sex did not demonstrate statistically significant differences between groups. These exploratory analyses collectively suggest that the causes of high heterogeneity may be more complex, and future research on CRCI should consider multiple variables.

Meanwhile, when focusing on studies that provide adjusted estimates (standardized regression coefficient *β*), the association between sleep disorders and cognitive impairment exhibited high robustness and consistency (*I*^2^ = 0.1%). This result stands in stark contrast to the analysis based on simple correlation coefficients *r* (*I*^2^ = 92.3%), uncovering the root cause of the initial high heterogeneity with the confounding effects of variables. This further explains why subgroup analysis and meta-regression failed to detect significant heterogeneity. The pooled *β* value of −0.28 suggests that poorer sleep quality is associated with greater cognitive impairment after accounting for potential confounders. Further supporting the meta-analysis results based on *r*-values in this study.

Although this study confirmed a significant correlation between sleep quality and CRCI in patients with cancer through meta-analysis, the following limitations remain. First, substantial heterogeneity between the included studies was detected, which could not be fully eliminated through subgroup analysis, and the exact source of heterogeneity remains unidentified. Future research should incorporate more diverse samples to improve comparability between studies. Second, patients with breast cancer predominated in the included studies, whereas samples from other types of cancer were relatively limited, potentially affecting the generalizability of the conclusions. Finally, since the reviewed literature primarily comprised cross-sectional studies in this study, the results can only establish an association between sleep quality and CRCI rather than inferring causality. Future research should incorporate longitudinal or experimental designs to enable causal inference.

## 5 Conclusion

This study found a moderate negative correlation between sleep quality and CRCI in patients with cancer. Multiple confounding factors collectively influenced the size of the effect, and the assessment tools used to measure CRCI may be one of the reasons for the high heterogeneity observed in this study. Future research is recommended to prioritize standardized cognitive assessment tools and perform stratified analyses across different types of cancer to further explore the relationship between sleep quality and CRCI.

## Data Availability

The original contributions presented in the study are included in the article/Supplementary material, further inquiries can be directed to the corresponding author.
