# Supplementary material for: Association between sleep quality and cancer-related cognitive impairment in patients with cancer: a meta-analysis
Source: Front Neurol. 2026 Mar 18;17:1768687. doi: 10.3389/fneur.2026.1768687 (PMC13038526; doi:10.3389/fneur.2026.1768687)
Supplement: Supplementary file 1 [file Table_1.docx]

Supplementary Material

**Table S1 Retrieval strategies and results for all databases**

| **Database name** | **Search results** | **Search strategy** |
| --- | --- | --- |
| Pubmed | 352 | ( **(Cognition[MeSH Terms]) OR ("Cognition Disorders"[Title/Abstract] OR "Cognitive Dysfunction"[Title/Abstract] OR "Cognitive Deficit"[Title/Abstract] OR "cognitive impairment"[Title/Abstract] OR "cognitive deficit"[Title/Abstract] OR "cognitive problem"[Title/Abstract] OR "cognitive defect"[Title/Abstract]))AND((Neoplasms[MeSH Terms]) OR ("Neoplasms"[Title/Abstract] OR "Carcinoma"[Title/Abstract] OR "cancer"[Title/Abstract]))AND((sleep[MeSH Terms]) OR ("Sleep Disorders"[Title/Abstract] OR "Insomnia"[Title/Abstract] OR "sleep disturbance"[Title/Abstract] OR "sleep disturb"[Title/Abstract] OR "sleep complaint"[Title/Abstract]))** |
| Scopus | 813 | ( TITLE-ABS-KEY ( "cognition disorders" or "cognitive dysfunction" or "cognitive deficit" or "cognitive impairment" or "cognitive deficit" or "cognitive problem" or "cognitive defect" ) and TITLE-ABS-KEY ( "neoplasms" or "carcinoma" or "cancer" ) and TITLE-ABS-KEY ( "sleep disorders" or "insomnia" or "sleep disturbance" or "sleep disturb" or "sleep complaint" ) ) and ( LIMIT-TO ( SRCTYPE , "j" ) ) and ( LIMIT-TO ( LANGUAGE , "english" ) or LIMIT-TO ( LANGUAGE , "chinese" ) ) and ( LIMIT-TO ( DOCTYPE , "ar" ) ) |
| Embase | 329 | ('cognition disorders':ti,ab,kw OR 'cognitive dysfunction':ti,ab,kw OR 'cognitive impairment':ti,ab,kw OR 'cognitive deficit':ti,ab,kw OR 'cognitive problem':ti,ab,kw OR 'cognitive defect':ti,ab,kw) AND ('neoplasms':ti,ab,kw OR 'carcinoma':ti,ab,kw OR 'cancer':ti,ab,kw) AND ('sleep disorders':ti,ab,kw OR 'insomnia':ti,ab,kw OR 'sleep disturbance':ti,ab,kw OR 'sleep disturb':ti,ab,kw OR 'sleep complaint':ti,ab,kw) |
| Web of scince | 215 | topic("Cognition Disorders" OR "Cognitive Dysfunction" OR "Cognitive Deficit" OR "cognitive impairment" OR "cognitive deficit" OR "cognitive problem" OR "cognitive defect")AND(“Neoplasms" OR "Carcinoma" OR "cancer" )AND( "Sleep Disorders" OR "Insomnia" OR "sleep disturbance" OR "sleep disturb" OR "sleep complaint") |
| CNKI | 212 | SU%=(cognitive impairment + cognitive deficit + cognitive change + cognitive alteration + cognitive function) AND SU%=(cancer + malignancy + tumor) AND SU%=(sleep quality + sleep disorder + sleep disturbance + sleep problem + insomnia) |
| Wanfang | 180 | ((Topic=cancer) OR Title/Keywords=(cancer OR tumor OR malignant tumor)) AND ((Topic=sleep disorder) OR Title/Keywords=(sleep quality OR sleep disorder OR sleep disturbance OR sleep problem OR insomnia)) AND ((Topic=cognitive impairment) OR Title/Keywords=(cognitive impairment OR cognitive deficit OR cognitive change OR cognitive alteration OR cognitive function)) |
| VIP | 663 | U=(cancer OR tumor) AND U=(sleep quality OR sleep disorder OR sleep disturbance OR sleep problem OR insomnia) AND U=(cognitive impairment OR cognitive deficit OR cognitive change OR cognitive alteration OR cognitive function) |
| SinoMed | 1273 | ( "cognitive impairment"[unweighted:expanded] OR "cognitive impairment"[common field:intelligent] OR "cognitive deficit"[common field:intelligent] OR "cognitive change"[common field:intelligent] OR "cognitive alteration"[common field:intelligent] OR "cognitive function"[common field:intelligent]) AND ( "neoplasms"[unweighted:expanded] OR "cancer"[common field:intelligent] OR "tumor"[common field:intelligent]) AND ( "sleep quality"[common field:intelligent] OR "sleep disorder"[common field:intelligent] OR "sleep disturbance"[common field:intelligent] OR "sleep problem"[common field:intelligent] OR "insomnia"[common field:intelligent]) |
