# Supplementary material for: Association between sleep quality and cancer-related cognitive impairment in patients with cancer: a meta-analysis
Source: Front Neurol. 2026 Mar 18;17:1768687. doi: 10.3389/fneur.2026.1768687 (PMC13038526; doi:10.3389/fneur.2026.1768687)
Supplement: Supplementary file 2 [file Table_2.docx]

Supplementary Material

**Table S2 Basic characteristics of included literature**

| First author/ year | Type of study | Region | Male/ female (ex.) | Mean age | *r*（*r_s_*）/*β* | Effect size statistical methods | Sleep measurement tools | Cognitive measurement tools | Diseases | Main Conclusions |
| --- | --- | --- | --- | --- | --- | --- | --- | --- | --- | --- |
| Wu (14) 2024 | Cross-sectional | United States | 0/81 | 56.8±12.10 | -0.071 | Spearman | PSQI | MoCA | Breast | Cognitive impairment was not directly associated with sleep disorders |
| Fang (15) 2016 | Cross-sectional | China | 49/31 | 23.77±9.75 | -0.390 | Pearson | PSQI | EORTC-CF | Bone cancer | Sleep disturbance was significantly negatively correlated with quality of life |
| Meng (16) 2020 | Cross-sectional | China | 3/352 | 52.31±10.45 | -0.485 | Pearson | PSQI | PRMQ | Breast | Sleep quality is significantly associated with memory |
| Henneghan (17) 2018 | Cross-sectional | United States | 0/90 | 49.00 | -0.400 | Pearson | PSQI | FACT-Cog | Breast | Significant moderate correlations were found between the FACT-Cog and PSQI subscales |
| Gu (18) 2025 | Cross-sectional | China | 53/27 | 55.47±6.23 | -0.442 | Pearson | PSQI | MMSE | Primary liver cancer | Sleep disorders aggravate pain, inflammation, and reduce cognitive function |
| Chen (19) 2024 | Cross-sectional | China | 0/294 | 54.69±10.49 | -0.750/  -0.245 | Pearson/multiple linear regression analysis | PSQI | FACT-Cog | Breast | Sleep quality has a direct negative effect on cognitive impairment |
| Zhang (20) 2020 | Cross-sectional | China | 0/188 | 67.80±4.90 | -0.577/  -0.354 | Pearson/multiple linear regression analysis | PSQI | FACT-Cog | Breast | Sleep disorders were significantly associated with cognitive impairment |
| Rogers (21) 2008 | Cross-sectional | United States | 43/15 | 60.40±13.10 | -0.410/  -0.320 | Spearman/multiple linear regression analysis | PSQI | FACT-Cog | Head and Neck cancer | Sleep disturbance was negatively associated with total cognitive function score |
| Liu (22) 2024 | Cross-sectional | China | 0/741 | 50.38±9.76 | -0.201 | Pearson | PSQI | FACT-Cog | Breast | PSQI total score was negatively correlated with FACT-Cog |
| Chen (23) 2008 | Cross-sectional | China | 74/41 | 59.40±10.70 | -0.550/  -0.309 | Pearson/multiple linear regression analysis | PSQI | EORTC-CF | Lung cancer | Sleep disorders independently predict cognitive decline |
| Hutchinson (24) 2021 | Cross-sectional | Australia | 15/15 | 50.80±13.20 | -0.460/  -0.160 | Pearson/multiple linear regression analysis | PSQI | FACT-Cog | Hematologic cancers | Sleep problems may indirectly affect cognitive function through fatigue |
| Xu (25) 2024 | Cross-sectional | China | 70/16 | 64.10±9.53 | -0.434/  -0.180 | Pearson/multiple linear regression analysis | PSQI | FACT-Cog | Lung cancer | Sleep quality is an independent influencing factor for cognitive impairment in lung cancer patients undergoing chemotherapy |
| Garland (26) 2022 | Prospective cohort | Canada | 252/458 | 57.00±9.90 | -0.330 | Pearson | ISI | CFQ | cancers | There is a bidirectional association between insomnia and perceived cognitive impairment. |

Note: MoCA: Montreal Cognitive Assessment; MMSE: Mini-Mental State Examination (MMSE); FACT-Cog: Functional Assessment of Cancer Therapy-Cognitive Function; PRMQ: Prospective and Retrospective Memory Questionnaire; CFQ: Cognitive Failures Questionnaire; EORTC-CF: European Organisation for Research and Treatment of Cancer-Cognitive Function (EORTC-CF); PSQI: Pittsburgh Sleep Quality Index (PSQI); ISI: Insomnia Severity Index.
